# Pulmonary Arterial Hypertension and Hereditary Haemorrhagic Telangiectasia

**DOI:** 10.3390/ijms19103203

**Published:** 2018-10-17

**Authors:** Veronique M. M. Vorselaars, Anna E. Hosman, Cornelis J. J. Westermann, Repke J. Snijder, Johannes J. Mager, Marie-Jose Goumans, Marco C. Post

**Affiliations:** 1 Department of Cardiology, St. Antonius Hospital, 3435 CM Nieuwegein, The Netherlands; m.post@antoniusziekenhuis.nl; 2 Department of Pulmonology, St. Antonius Hospital, 3435 CM Nieuwegein, The Netherlands; a.hosman@antoniusziekenhuis.nl (A.E.H.); cjjw@xs4all.nl (C.J.J.W.); r.snijder@antoniusziekenhuis.nl (R.J.S.); j.mager@antoniusziekenhuis.nl (J.J.M.); 3 Department of Molecular Cell Biology, Leiden University Medical Centre, 2333 ZA Leiden, The Netherlands; M.J.T.H.Goumans@lumc.nl

**Keywords:** pulmonary arterial hypertension, hereditary haemorrhagic telangiectasia, pulmonary hypertension, *ACVRL1*, *ENG*

## Abstract

Hereditary haemorrhagic telangiectasia (HHT) is an autosomal dominant inherited disease characterised by multisystemic vascular dysplasia. Heritable pulmonary arterial hypertension (HPAH) is a rare but severe complication of HHT. Both diseases can be the result of genetic mutations in *ACVLR1* and *ENG* encoding for proteins involved in the transforming growth factor-beta (TGF-β) superfamily, a signalling pathway that is essential for angiogenesis. Changes within this pathway can lead to both the proliferative vasculopathy of HPAH and arteriovenous malformations seen in HHT. Clinical signs of the disease combination may not be specific but early diagnosis is important for appropriate treatment. This review describes the molecular mechanism and management of HPAH and HHT.

## 1. Pulmonary Hypertension

Pulmonary hypertension (PH) is a complex pathophysiological and haemodynamic condition which can complicate many cardiovascular and respiratory diseases. It is defined as a mean pulmonary arterial pressure (PAP) of ≥25 mmHg at rest, measured with right heart catheterisation (RHC). PH can be classified in five groups based on a clinical classification with (partly) similar clinical presentation, pathological finding, haemodynamic characteristics, and treatment options: group (I) Pulmonary arterial hypertension (PAH); group (II) PH due to left heart disease; group (III) PH due to lung diseases and/or hypoxemia; group (IV) chronic thromboembolic pulmonary hypertension; and group (V) PH with unclear/multifactorial origin [1]. Treatment differs per subgroup and may be directed to the underlying cause. This review has the focus on PH (mainly PAH) and hereditary haemorrhagic telangiectasia (HHT). We will discuss both diseases, their overlapping molecular mechanisms, and management strategies.

## 2. Pulmonary Arterial Hypertension

PAH is a rare (15–25 cases per million persons), but severe vascular disorder with increased PAP as a result of vascular remodelling of the pulmonary circulation [1]. Proliferation of endothelial cells (ECs) and vascular smooth muscle cells (SMCs) reduce the intraluminal space of the pulmonary arterioles thereby increasing the pulmonary arterial pressure, eventually leading to right ventricular failure.

PAH is defined by an increased mean PAP (of ≥25 mmHg at rest), a pulmonary capillary wedge pressure (PAWP) ≤ 15 mmHg and an increased pulmonary vascular resistance (PVR) of >3 Wood units, for all of which the gold standard of measurement is a RHC. However, an echocardiogram can also give an estimation of the severity of the disease estimating right ventricular pressure and evaluating secondary signs of right ventricle overload [1].

Clinical features are the result of decrease in cardiac output due to right heart failure and include progressive dyspnoea, decreased exercise tolerance, (near-) collapse, chest pains, oedema, and fatigue. Since the symptoms are aspecific, diagnosing can be challenging and a significant delay can occur [1].

PAH is associated with several conditions and factors. Firstly, specific drugs have been associated with the development of PAH; these mainly include anorexigens, although methamphetamines have also been linked to PAH. Secondly, congenital heart disease causing a left-to-right shunt may lead to reversal of the shunt due to an increase in PVR leading to Eisenmenger syndrome. Additionally, connective tissue disease (mainly systemic sclerosis), human immunodeficiency virus, and portal hypertension can result in PAH.

The diagnosis heritable PAH (HPAH) is applicable when one of several genetic mutations is found. Mutations in the bone morphogenetic protein receptor 2 (*BMPR2*) are found in 75% of patients in this category, of which the penetrance is approximately 30% [2,3,4]. However, in the remaining 25% of patients other associated genetic mutations are found. These include *ACVLR1*, *ENG*, *SMAD4*, and *BMP9* (also known as *GDF2*), of which the first three are also associated with HHT [5,6]. These genes all encode for proteins that play a role in the transforming growth factor-beta (TGF-β) superfamily signalling pathway. In some families with HPAH no pathologic mutation is found [2]. Idiopathic PAH (IPAH) is a diagnosis per exclusionem when no underlying cause is found.

## 3. Hereditary Haemorrhagic Telangiectasia

HHT, also known as Rendu–Osler–Weber disease (ROW), is an autosomal dominant inherited disease with estimated worldwide prevalence is around 1 in 5000 individuals, although large regional variance exists. Multisystemic vascular dysplasia results in mucocutaneous telangiectasia (focal dilatation of postcapillary veins) and arteriovenous malformations (AVMs). AVMs can theoretically grow in every organ, but most frequent effected organs are the lung, brain, and liver. These AVMs are susceptible to rupture and haemorrhage, leading to major morbidity and mortality.

Pulmonary AVMs result in a direct blood flow from the pulmonary artery to the pulmonary vein, bypassing the capillary–alveolar barrier without effective gas exchange and resulting in a reduced filtering capacity of the pulmonary capillary bed. Complications from pulmonary AVMs therefore mainly include hypoxemia and paradoxal (sterile or septic) emboli, although many patients remain asymptomatic. A contrast echocardiogram can be used to screen for pulmonary AVMs in HHT patients [7]. To reduce the risk of these severe complications, patients should be treated with embolisation; an endovascular intervention that occludes the feeding artery of the pulmonary AVM with vascular plugs or coils [8].

Hepatic shunting as a result of vascular malformation is present in 32 to 78% of HHT patients and occurs in three different types: shunting from hepatic artery to portal vein, hepatic artery to hepatic vein, and/or portal vein to hepatic vein. It can lead to portal hypertension, biliary necrosis, and high output cardiac failure due at least two- to three-fold increase in cardiac output [9,10].

Complications of cerebral AVMs are rare (approximately 0.5% per year) but its consequences can be devastating [11].

Not only dilation of the vascular lumen but also thinning of the vascular wall characterizes telangiectatic lesions. As these telangiectasia often occur in the nasal mucosa, the most prominent clinical feature of HHT is epistaxis [12], 96% of patients with HHT suffer from epistaxis, of which more than 50% before the age of 20 [13]. Both epistaxis and especially gastrointestinal bleeding can lead to severe anaemia [14].

The majority of cases are caused by mutations in the *ENG* (cytogenetic location 9q34.11; OMIM187300, encoding for the Endoglin protein) or *ACVRL1* genes (cytogenetic location 12q13.13; OMIM600376, encoding for the ALK1 protein). These mutations result in a haploinsufficiency with reduced levels of functional proteins of Endoglin and Activin receptor like kinase 1 (ALK1), respectively, and can be found in up to 95.7% of HHT patients [15]. A third disease-causing mutation has been found in the *SMAD4* gene (cytogenetic location 18q21.2; OMIM175050), resulting in a combination of juvenile polyposis syndrome and HHT [16]. This mutation is only found in 1 to 2% of HHT patients. All types of mutations have been reported, including missense, nonsense, deletions, insertions, and splice site. Most families with HHT have a unique mutation and more than 900 mutations are described [17]. *ENG* mutations cause HHT type 1 which is characterised by a higher prevalence of pulmonary and cerebral AVMs, mucocutaneous telangiectasia, and epistaxis compared to *ACVRL1* mutations, or HHT type 2. The second has a higher prevalence of hepatic AVMs.

The diagnosis is based on genetic testing or on the Curacao criteria. These criteria include (1) recurrent and spontaneous epistaxis; (2) visceral localisation; (3) an affected first-degree family member; and (4) the presence of mucocutaneous telangiectases. When an individual shows three or more criteria, they are considered to have HHT. When they meet two criteria the diagnosis is possible and with one or none criteria, HHT is considered unlikely [12].

Patients with HHT type 1, especially women who have not been screened and treated preemptively, have a slightly lower life expectancy than family members without HHT and severe epistaxis can result in a decreased quality of life [18]. However, preliminary data show that a normal life expectancy can be achieved when patients are screened and treated appropriately [19].

In HHT most symptoms are progressive with age. Clinical signs are not only variable in subtype and age but also variable in severity between family members with identical mutations [20]. Etiological factors and genetic modifiers are thought to explain this clinical variability [21,22].

## 4. Molecular Mechanism

**Figure 1:**
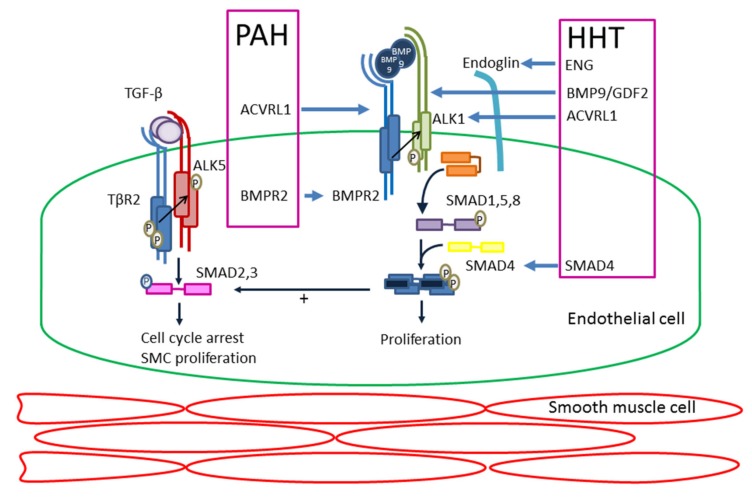
Schematic diagram illustrating the transforming growth factor-beta (TGF-β) pathway and the genes and proteins involved in pulmonary arterial hypertension (PAH) and hereditary haemorrhagic telangiectasia (HHT). Illustrated are two pathways of ALK5/SMAD2-3 and ALK1/SMAD1-5. SMC, smooth muscle cell. P, phosphorylation.

The TGF-β superfamily signalling pathway has been recognised to play an important role in different cellular processes including proliferation, migration and apoptosis [23]. The TGF-β is a complex pathway, which plays a pivotal role in the process of angiogenesis using two distinct signalling pathways: the activin receptor-like kinase 5 (ALK5)-Smad2/3 pathway and the ALK1-Smad1/5/8 pathway [24,25] (Figure 1). Although much research has been done on the effects of ALK1, its role in angiogenesis has been shown inconsistent [26,27,28]. When vessels are formed ECs migrate and proliferate. Once the capillary wall is formed, pericytes help stabilise the vessel and inhibit EC proliferation and migration. This leads to vascular maturation, a process in which ALK5 plays an important role. Endoglin is upregulated by ALK1 and is an accessory receptor in the TGF-β signalling pathway, which is particularly expressed on proliferating ECs [29]. It has been found that endoglin counterbalances the stabilizing role of ALK5 [30]. Mutations in *ENG* and *ACVRL1* genes disrupt TGF-β signalling, altering EC tubulogenesis and pericyte recruitment causing abnormal capillary formation and maturation leading to venous enlargement, vascular hyperbranching, and arteriovenous malformations explaining the abnormal morphogenesis of vasculature in HHT [24,31].

EC also regulate vascular function by controlling the production of vasoconstrictors, vasodilators and the activation and inhibition of SMCs. Disruption of the SMAD1/5/8 pathway and BMP signalling, as a consequence of a *BMPR2* or *ACVRL1* mutation, results in inhibition of apoptosis of SMC leading to SMC proliferation and vascular remodelling, ultimately causing PAH [32,33,34]. Interestingly, both PAH and HHT originate in defects in the BMP9/ALK1/Endoglin pathway (Figure 1). BMPR2 forms a signalling complex with ALK1, which responds to BMP9 by binding with high affinity to ALK1 and Endoglin [5,35]. A case report has shown that a mutation in *BMP9* can lead to a syndrome with phenotypic similarities with HHT [36]. Recently, BMP9 has been used in animal studies to treat PAH by stimulating BMPR2 signalling [37,38]. Hypothetically it might be possible that BMP9 treatment has a therapeutic effect on HHT.

## 5. Heritable Pulmonary Arterial Hypertension (PAH) and Hereditary Haemorrhagic Telangiectasia (HHT)

HHT can be complicated by HPAH, although this is a rare complication. *ACVRL1* mutations have been recognised to lead to this combined syndrome for several years. In total, 79 patients with PAH and *ACVRL1* mutations have been described in the literature (Table 1) [39,40,41,42,43,44,45,46,47,48,49]. This includes both patients with HPAH with clinical features of HHT and patients diagnosed with HHT who develop HPAH. In many of these patients, PAH was diagnosed before the clinical symptoms of HHT became manifest. Many different *ACVRL1* mutations have been described in HPAH patients, but there seems to be a predominance of mutations in exon 10 and particularly in the nonactivating non-downregulating (NANDOR) box [17]. However, most family members of HHT patients with HPAH will not develop HPAH, which indicates that additional genetic or environmental factors are necessary to develop the HPAH phenotype [44].

Knowledge of the disease combination of PAH and HHT is especially important since this combination usually leads to a worse outcome than PAH alone [44]. Twenty-two of the patients described in these case reports were diagnosed under the age of 18 (28%). Compared to *BMPR2* mutation carriers and noncarriers (idiopathic PAH), *ACVRL1* mutation carriers are diagnosed at a younger age and have a worse prognosis despite similar therapy and better haemodynamics at time of diagnosis [44]. This suggests that the disease progresses more rapidly with severe consequences. A similar study by Li et al. [57] compared nine HHT-PAH (mutation unknown) patients to 18 IPAH patients, evaluating their prognosis. One- and three-year survival rates were 78% and 53% for HHT-PAH patients, respectively; significantly lower than patients with IPAH (one- and three-year survival 91% and 74%, *p* = 0.047) [57].

Eight patients with HPAH and HHT resulting of *ENG* mutations have been described in literature (Table 1) [42,49,50,52,53]. However, one patient had exposure to dexfenfluramine that may be the leading cause of PAH. No data exist about the prognosis of patients with PAH and *ENG* mutations, although it can be expected to be lower compared to *ENG* patients without HPAH.

The role of *SMAD4* in the pathogenesis of HPAH is not completely revealed. Although there are no HHT related *SMAD4* mutation carriers described with HPAH, there are two PAH patients in whom a mutation in the *SMAD4* gene is found [49].

Even though it is rare for HHT to be complicated by PAH, physicians should be aware of the combination and perform an echocardiogram when clinical signs indicate so, especially in patients with *ACVRL1* mutations. Conversely, clinical signs of HHT in patients with HPAH based on *ACVRL1* mutations might not always be apparent initially due to less severe phenotype of HHT type 2.

In both diseases differences are seen between men and women. Epidemiologic data shows a female predominance in many types of PAH and life expectancy of females with HHT caused by an *ENG* mutation seems to be impacted greatly [18,59]. Although it is thought female hormones play an important role in both diseases, the exact mechanisms are not yet fully understood [60,61,62].

This review focuses on patients with the disease combination HHT and HPAH. However, there are more cases with reported *ENG* or *ACVRL1* pathologic variants and HPAH but no clinical signs of HHT [49]. Interestingly in some of these cases, family members with the same DNA variant show no signs of PAH or HHT [63,64]. Data on the prevalence are not available.

## 6. Diagnosis of Pulmonary (Arterial) Hypertension in Hereditary Haemorrhagic Telangiectasia (HHT)

**Figure 2:**
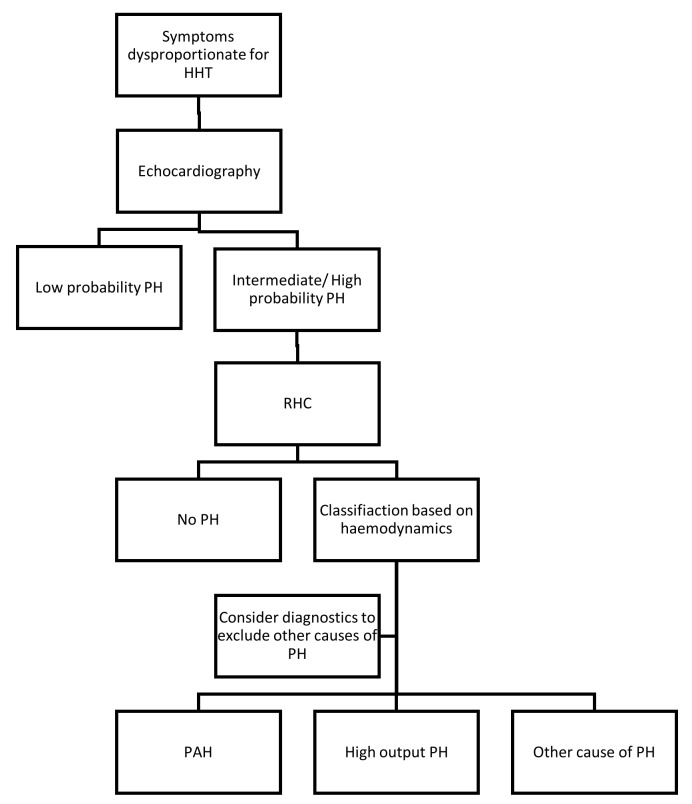
Flow chart diagnosis of PH in HHT. HHT, hereditary haemorrhagic telangiectasia; PH, pulmonary hypertension; RHC, right heart catheterisation; PAH, pulmonary arterial hypertension.

Differentiating between common symptoms of HHT and HHT complicated by PAH can be challenging. Patients with HHT often suffer from fatigue, shortness of breath, and exercise intolerance due to anaemia, hypoxaemia as a result of pulmonary AVMs, disturbed sleep due to epistaxis, and the psychological strain of a chronic disease. The diagnostic management of PH in HHT depends on the presence of symptoms (Figure 2). When a patient’s history or physical examination suggests PH (e.g., dyspnoea without presence, or discrepant to the level of anaemia, or large pulmonary AVMs), an echocardiogram should be performed to assess the probability of PH.

Echocardiography can estimate systolic PAP from the right atrium to right ventricle pressure gradient based on the peak tricuspid regurgitation velocity (TRV) in combination with secondary echocardiographic signs of PH. Category A: an increased right ventricle/left ventricle ratio, flattening of the interventricular septum; category B: increase diameter of the pulmonary artery, short right ventricular outflow Doppler acceleration time, and increased early diastolic pulmonary regurgitation velocity; category C: dilation of the inferior vena cava or right atrial dilation. Based on these characteristics on echocardiography the probability of the presence of PH can be classified as low, intermediate, or high. Low probability is defined as peak TRV ≤ 2.8 m/s without secondary signs, intermediate probability as peak TRV ≤ 2.8 m/s with secondary signs (at least of two different categories) or peak TRV 2.9–3.4 m/s without secondary signs, and high probability peak TRV 2.9–3.4 m/s with secondary signs or peak TRV > 3.4 m/s. In case of intermediate or high probability, further investigation with RHC should be considered to confirm the diagnosis [1]. Characteristic haemodynamics in patients with HPAH are (1) a more pronounced increase in mean PAP, (2) high PVR, and (3) low PAWP (Table 2). Computed tomography, ventilation/perfusion lung scan, a pulmonary function test, and blood test should be performed to exclude other causes of PH [1].

Echocardiography is used to screen for pulmonary AVMs and may show an intermediate or high probability of PH in asymptomatic patients. Since there is no therapeutic consequence in respect to elevated pulmonary pressures in truly asymptomatic patients a RHC is not recommended. No exact recommendations for follow-up are described in the guidelines, but monitoring of symptoms seems indicated. When symptoms arise echocardiography should be performed.

## 7. Diagnosis of Hereditary Haemorrhagic Telangiectasia (HHT) in Heritable Pulmonary Arterial Hypertension (HPAH)

**Figure 3:**
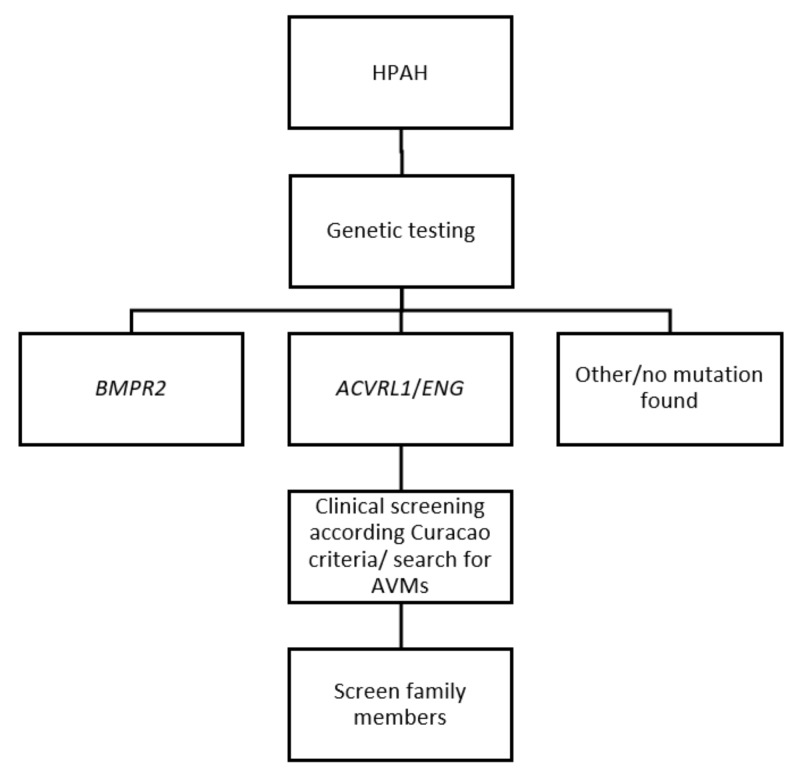
Diagnosis of HHT in HPAH. HPAH, heritable pulmonary arterial hypertension; AVMs, arteriovenous malformations.

Genetic testing is advised in all patients suspected for HPAH (meaning PAH patients with no known cause). When no *BMPR2* mutation is identified, screening for *ACVRL1* or *ENG* mutations should be considered (Figure 3) [1]. Conversely, genetic testing in asymptomatic patients should not be taken lightly, making expert counselling of a geneticist indispensable. The lifetime risk of developing PAH is approximately 20% in patients with a *BMPR2* mutation [66]. Although a negative test for the mutation can be reassuring, a positive test can give a great psychological strain. Furthermore, as mentioned above, it is not certain how to screen asymptomatic patients and there is no evidence whether targeted therapy in asymptomatic patients is beneficial.

Patients with HPAH that has resulted from *ACVLR1*, *ENG*, and *BMP9* mutations could be affected by HHT. It can be debated whether a patient with a pathological *ACVLR1*, *ENG*, and *BMP9* mutation, that has led to HPAH and is known to cause HHT, per definition also has HHT based on genetics and clinical features are not yet visible, or that those patients may not develop HHT at all. It should be considered though that some of these PAH patients have little symptoms of HHT but can still be affected by silent AVMs. Therefore, screening for AVMs should be performed to prevent future complications. When patients are considered to have HHT it is recommended to screen all first-line family members.

## 8. Management of Heritable Pulmonary Arterial Hypertension (PAH) in Hereditary Haemorrhagic Telangiectasia (HHT)

Although examples in the literature are limited, treatment with the typical therapies used for HPAH is recommended. This includes a combination of different PAH-specific medication (endothelin receptor antagonists (ERA), phosphodiesterase inhibitors (PD5I), soluble guanylate cyclase stimulators, and prostacyclins) which cause vasodilation of the pulmonary vasculature and supporting therapy (e.g., diuretics, oxygen, salt reduction, iron substitution, rehabilitation, and psychological help) [1]. The aim of treatment is reducing pulmonary arterial pressure and reducing symptoms (increase exercise capacity and quality of life). General recommendations are to avoid excessive exercise and pregnancy. Oral anticoagulation is advised for HPAH and IPAH patients, but the increased bleeding tendency in HHT makes treatment with oral anticoagulation difficult [67]. Although there is no absolute contraindication, many patients will not tolerate anticoagulation. These treatments should be performed by HHT or PAH specialist in tertiary referral centres [1].

Several different treatment regimens are described but no large randomised trials exist. Intensity of treatment is based on severity of symptoms, which can be objectified by performing a 6-min walking test. Two case reports describe successful treatment with the ERA bosentan in PAH and HHT, which improves exercise capacity, laboratory findings, and hemodynamic parameters [68,69]. Recently the first case of a patient successfully treated with sildenafil (PD5I) was documented [55]. Tadalafil (PD5I) and ambrisentan (ERA) are also described in a severe case of HPAH, although unfortunately not successful [58].

Vasoreactivity testing with vasodilators with a short duration of action (such as inhaled nitric oxide) is recommended in all patients with HPAH to detect patients suitable for treatment with calcium channel blockers. Although, there was no reaction on pulmonary vasodilators in a study with 23 *ACVRL1* patients, Ca-blockers should nevertheless be tested in HHT [1,44]. The effect can be dramatic in PAH if vasoresponders are identified.

In more severe cases with refractory symptoms despite optimal therapy (including a combination of three different PAH medications and supporting therapy with diuretics and oxygen), lung transplantation may be a last option. Smoot et al. describes a young HHT patients with severe HPAH (mean PAP 67 mmHg) successfully treated with a bilateral lung transplantation [43].

It is important to realise that embolisation of pulmonary AVMs could potentially increase the pulmonary arterial pressure due to closure of a low-resistance pathway, although to which extent this might contribute to the progression of PH is not yet known [70,71,72,73]. Shovlin et al. [70] found no significant increase in mean PAP after embolisation in patients with mild to moderate PH. A possible explanation is the decrease in CO after embolisation (with a greater effect on the PVR) than the occlusion of the pulmonary AVM(s) or the pulmonary AVM related hypoxemia (with concomitant vasoconstriction and therefore an increase in PVR) [71]. Furthermore, the risk of sudden rupture of pulmonary AVMs may be increased in PAH patients [46]. Management strategies should therefore be made on a case-by-case basis (depending on size of pulmonaryAVM and severity of PH).

## 9. Future Therapies

Although local treatment of telangiectasia and pulmonary AVMs are increasingly successful, a good systemic therapy is not yet available. Multiple studies and trials are being performed researching potential drug targets and the possibilities of repurposing existing drugs.

Tacrolimus, a drug used in the prevention of the rejection of an allogeneic organ transplant, has been investigated as a *BMPR2* activator in HHT. It has been found to be effective in vitro and in vivo (mouse models) decreasing the incidence of AVMs. Tacrolimus increases endoglin and ALK1 expression [74]. It activates Smad1/5/8 and opposes the proangiogenic gene expression signature associated with ALK1 loss-of-function seen in HHT type II [75]. The implications of Tacrolimus in PAH have also been studied and show that it can reverse severe PAH in rats. A handful of case reports on patients with severe IPAH treated with tacrolimus show promising results with reductions of the NYHA (New York Heart Association) class [76].

Bevacizumab, a humanised monoclonal antibody (IgG1) used in the treatment of various cancers, is viewed as a potential therapy for HHT. The antibody is directed to vascular endothelial growth factor, inhibiting neoangiogenesis. Although topical use of Bevacizumab to treat epistaxis has not shown to be beneficial in different randomised control trials, intravenous and submucosal use seems to reduce epistaxis and gastro-intestinal bleeding, but further studies need to be performed to confirm these results [77,78].

Very recently, octreotides have been suggested to have a possible positive effect on clinical symptoms of HHT [79]. Octreotides are an analogue of natural somatostatine, inhibiting growth hormones and insulin like growth factor 1 and is used in gastrointestinal neoplasms and neuroendocrine tumours. Although the underlying mechanism in relation to relieving the symptoms of HHT has not yet been investigated, currently clinical trials are being undertaken.

Thalidomide, also known as Softenon, has shown to effectively reduce epistaxis by promoting vessel maturation. However, most patients suffer from side effects in time to severe to continue the therapy (including neuropathy, severe skin reactions, angina and dyspnoea, oedema, drowsiness, general malaise, and tremor) [58,80].

## 10. Pulmonary Hypertension as Complications of Hereditary Haemorrhagic Telangiectasia (HHT)

This review discusses the combination of PAH and HHT particularly, but it is important to note that other types of PH, associated with HHT, can occur by several different mechanisms. This often involves PH due to left sided heart disease or high output PH due to a left-to-right shunt in the presence of AVMs in the liver resulting in a hyperkinetic state [45,81,82]. Increase in cardiac output leads to an elevation in mean PAP (estimated increase in mean PAP up to 0.5 to 3.0 mmHg per litre/min increase in cardiac output) [83]. Especially in HHT, anaemia due to epistaxis and gastro-intestinal bleeding may trigger this cascade due to increased cardiac output. Precapillary PH may be the result of chronic thromboembolic PH (CTEPH) since HHT patients may encounter an increased thrombotic risk [84]. Furthermore, all other forms of PH, not related to HHT, could exist in HHT patients as well. The overall occurrence of echocardiographic-based suspected PH in unselected HHT patients is found between 4% and 20% [45,54,56,81]. We previously described that RHC is indispensable in symptomatic cases since subclassification of PH is based on invasive measurement of haemodynamics. Vorselaars et al. described that age, hepatic AVMs and the *ACVRL1* mutation are predictors for an increased TRV in HHT patients [54].

Any of these types of PH in combination with HHT can lead to a worse prognosis. Chizinga et al. studied 651 HHT patients of whom 13% had PH defined as a mean PAP > 25 mmHg during RHC [85]. Although the type of PH is not further defined in most of these patients, there is a significant associated mortality with PH in HHT patients (hazard ratio (adjusted for age) 3.8; *p* < 0.0001) [85].

## 11. Conclusions

The combination of HPAH and HHT is rare but may have severe consequences. Both diseases can be the result of mutations affecting the TGF-β signalling pathway, essential for angiogenesis. Clinical signs may not be specific but early diagnosis is important for appropriate treatment and prognosis. Therefore, awareness of this disease combination is important for all clinicians working with HHT or PAH patients 

## Figures and Tables

**Table 1:** PAH-HHT patients described in literature.

Study	Number of Patients	Genetics	Family Members
Trembath et al., 2001 [39]	8	*ACVRL1 n* = 8	*n* = 5 (2 families)
Harrison et al., 2003 [40]	14	*ACVRL1 n* = 9, *ENG n* = 2, Unknown *n* = 3 *	*n* = 0
Abdalla et al., 2004 [41]	10	*ACVRL1 n* = 10	*n* = 0
Chaouat et al., 2004 ^¥^ [50]	1	*ENG n* = 1 **	NA
Harrison et al., 2005 [42]	2	*ACVRL1 n* = 1, *ENG n* = 1	NA
Mache et al., 2008 ^¥^ [51]	1	*ENG n* = 1	NA
Smoot et al., 2009 [43]	3	*ACVRL1 n* = 3	*n* = 0
Girerd et al., 2010 [44]	9	*ACVRL1 n* = 9	*n* = 4 (1 family)
Lyle et al., 2015 [45]	12	*ACVRL1 n* = 4, Unknown *n* = 8 *	Unknown
Montani et al., 2009 ^¥^ [46]	1	*ACVRL1 n* = 1	NA
Chida et al., 2012 [47]	7	*ACVRL1 n* = 7	*n* = 0
Fujiwara et al., 2008 [48]	5	*ACVRL1 n* = 5	*n* = 0
Chen et al., 2013 [52]	12	*ACVRL1 n* = 7, *ENG n* = 2, Unknown *n* = 3 *	*n* = 0
Machado et al., 2015 [49]	1	*ACVRL1 n* = 1	NA
Girerd et al., 2016 [53]	10	*ACVRL1 n* = 9, *ENG n* = 1 ***	*n* = 2
Vorselaars et al., 2017 [54]	2	*ACVRL1 n* = 2	*n* = 0
Miyake et al., 2016 ^¥^ [55]	1	*ACVRL1 n* = 1	NA
Revuz et al., 2017 [56]	4	*ACVRL1 n* = 4	Unknown
Li et al., 2018 [57]	9	Unknown *n* = 9 *	Unknown
Nakamura et al., 2018 ^¥^ [58]	1	*ACVRL1 n* = 1	NA

^¥^ Case report, * based on Curacao criteria, ** exposure to dexfenfluramine, *** or signs or family history of HHT. NA, not applicable.

**Table 2:** Haemodynamics in pulmonary hypertension associated with hereditary haemorrhagic telangiectasia.

Haemodynamics	PAH	High Output PH
Mpap (mmHg)	++	+
PAWP (mmHg)	= (≤ 15)	=/+
PVR (Wood units)	++ (> 3)	=
CO (L/min)	−	++
DPG (mmHg)		− (< 7)

PH, pulmonary hypertension; HPAH, heritable pulmonary arterial hypertension; mPAP, mean pulmonary artery pressure; PAWP, pulmonary artery wedge pressure; PVR, pulmonary vascular resistance; CO, cardiac output; DPG, diastolic pressure gradient (diastolic PAP—mean PAWP). +, increased; =, normal; −, decreased; ++, severely increased. Adapted from Faughnan et al. [65], with the permission of the publisher.
